# Total ginsenosides enhance γ-globin expression and fetal hemoglobin production in β-thalassemia models

**DOI:** 10.3389/fphar.2025.1578237

**Published:** 2025-08-21

**Authors:** Dongling Cai, Ying Chan, Guangyu He, Yamin Kong, Aiqi Cai, Yan Guo, Baosheng Zhu

**Affiliations:** ^1^ Department of Medical Genetics, NHC Key Laboratory of Healthy Birth and Birth Defect Prevention in Western China, The First People’s Hospital of Yunnan Province, Kunming, China; ^2^ School of Medicine, Kunming University of Science and Technology, Kunming, China; ^3^ School of Life Sciences, Kunming University of Science and Technology, Kunming, Yunnan, China

**Keywords:** β-thalassemia, fetal hemoglobin, reactivation, γ-globin gene, total ginsenosides

## Abstract

**Introduction:**

β-thalassemia is a genetic hemoglobinopathy characterized by defective β-globin synthesis and ineffective erythropoiesis. Pharmacological induction of fetal hemoglobin (HbF) via γ-globin gene activation represents a promising therapeutic strategy. Total ginsenosides (TG), the principal active constituents of *Panax ginseng*, have shown epigenetic and transcriptional modulatory properties, yet their role in HbF induction remains unexplored.

**Methods:**

We evaluated the HbF-inducing potential of TG using human erythroleukemia cell line (K562), primary erythroid precursor cells (ErPCs) derived from CD34^+^ umbilical cord blood, and Townes transgenic mice. TG was administered at varying concentrations in vitro (25–400 μg/mL) and in vivo (50–800 mg/kg/day for 14 days). HbF and γ-globin expression were quantified by flow cytometry, immunofluorescence, and RT-qPCR. Hemoglobin content, cell viability, and hepatic histology were also assessed.

**Results:**

TG significantly induced HbF production and γ-globin gene expression in both cellular models in a dose-dependent manner. In K562 cells, 200 μg/mL TG elevated γ-globin mRNA by 4.29-fold; in ErPCs, the increase was 1.46-fold. HbF-positive cell populations rose markedly without impairing cell viability or morphology. In vivo, TG treatment at 200 and 400 mg/kg led to 2.8- and 3.1-fold increases in F-cell proportions, respectively, surpassing hydroxyurea controls. No hepatotoxicity was observed upon histopathological examination.

**Discussion:**

These findings establish TG as a potent, well-tolerated inducer of HbF through transcriptional activation of the γ-globin gene. Its efficacy across erythroid cell lines, primary progenitor cells, and transgenic mouse models underscores its translational potential as a natural therapeutic agent for β-thalassemia.

## 1 Introduction

β-thalassemia is an autosomal recessive hemoglobinopathy characterized by diminished or absent β-globin production (Origa, 2017), leading to chronic hemolytic anemia and cellular hypoxia (Kattamis et al., 2022). Mutations in the β-globin gene result in impaired synthesis of the β-globin peptide, which causes excessive α-globin peptide aggregation within red blood cells (Keith et al., 2023). This aggregation compromises the stability of the erythrocyte membrane and reduces the lifespan of the cells (Mohandas and Gallagher, 2008). Clinical manifestations of β-thalassemia encompass chronic anemia, iron overload, hepatosplenomegaly, cardiac insufficiency, and heart failure (Rachmilewitz and Giardina, 2011; Taher et al., 2018). The severity of symptoms correlates with the extent of α- and β-globin peptide chain imbalance (Mettananda et al., 2017). Phenotypes span from asymptomatic thalassemia traits to severe transfusion-dependent thalassemia that requires regular blood transfusions for survival (Thompson et al., 2018).

Fetal hemoglobin (HbF) plays a crucial role in reducing the severity of β-thalassemia by forming tetramers composed of 2 α-globin peptides and 2 γ-globin peptides (Bauer et al., 2012; Pavan et al., 2022). This prevents the aggregation of excessive α-globin peptide chains into harmful oligomers (Natta et al., 1974; Iftikhar et al., 2022). By carrying and delivering oxygen, HbF relieves the symptoms of β-thalassemia (Wu et al., 2019). However, currently available HbF inducers, such as 5-azacytidine (Clegg et al., 1983), decitabine (Olivieri et al., 2011), hydroxyurea (HU) (Algiraigri et al., 2017), butyrates (Rund and Rachmilewitz, 2000), and short-chain fatty acids (Pace et al., 2002), exhibit variable response magnitudes and are associated with side effects (Costa et al., 2022). Consequently, researchers are investigating new HbF inducers with improved safety and efficacy profiles. These include pharmacological agents and bioactive compounds derived from natural sources, such as camptothecin (Wang et al., 2015), resveratrol (Franco et al., 2014), curcumin (Koonyosying et al., 2020; Eghbali et al., 2023), cucurbitacin D (Liu et al., 2010) and angelicin (Viola et al., 2008), for the effective management of β-thalassemia.

Ginseng (Panax ginseng), a perennial herbaceous plant in the Araliaceae family (Guo et al., 2021), is highly valued in Asia for its medicinal properties, with a history spanning centuries (Fan et al., 2023). It is known for its various health benefits (Xu et al., 2017), such as relieving cold symptoms (Alsayari et al., 2021), providing energy (Bach et al., 2016), and promoting overall vitality (Wee et al., 2011). The primary active constituents of ginseng are the total ginsenosides (TG), which consist of a diverse range of steroidal saponins (Yu et al., 2021; Liu et al., 2024). Although TG’s positive effects on ischemia/reperfusion injury are well-established (Chen et al., 2023), their potential to stimulate γ-globin chain synthesis and their therapeutic implications have not been thoroughly explored. Recent findings suggest that ginsenosides have the ability to upregulate HBG gene expression and activate relevant pathways involved in γ-globin chain biosynthesis (Das et al., 2019). This insight is supported by microRNA and gene expression analysis, opening up new possibilities for research in this field.

In this study, we utilize human erythroleukemia cell line (K562) (Khan et al., 2020) and erythroid precursor cells (ErPCs) (Zeuner et al., 2003; Zuccato et al., 2021) as *in vitro* models, and Townes model mice as *in vivo* models (Woodard et al., 2022), to investigate the potential of TG to induce γ-globin gene expression and HbF production. Our objective is to validate TG as a promising therapeutic agent for the treatment of β-thalassemia.

## 2 Materials and methods

### 2.1 High-performance liquid chromatography analysis of TG

Ginsenoside standards, including ginsenosides Rg1, Re, Rf, Rg2, Rb1, Rc, Rb2, and Rd, were obtained from Yuanye Bio-Technology (Shanghai, China) and were dissolved in methanol for analysis. High-performance liquid chromatography (HPLC) evaluation was performed using a Waters Acquity UPLC H-Class system equipped with a Waters BEH C18 1.7 μm 2.1 × 100 mm column. The elution was carried out using a mobile phase composed of acetonitrile (A) and water with 1 mL of 85% phosphoric acid per liter (B). The gradient program of elution consisted of the following steps at a flow rate of 0.4 mL/min: starting with 19.0% A and gradually increasing to 21.0% at 7.31 min, 28.0% at 10.31 min, 31.0% at 16.31 min, and 38.5% at 21.31 min, reaching a peak of 90.0% at 22.00 min, and then decreasing to 19.0% at 25.50 min and maintaining that level until 27.50 min. Throughout the analysis, the column temperature was held constant at 45°C, and the absorbance was continuously monitored at 203 nm.

### 2.2 Culture and treatment of K562 cells

The K562 human chronic myelogenous leukemia cell line (Kunming Cell Bank, Kunming, China, ID: KCB90029YJ) was cultured in RPMI-1640 medium supplemented with 10% fetal bovine serum (FBS), 100 U/mL penicillin, and 100 mg/mL streptomycin, all obtained from Gibco (Grand Island, NY, United States) (Fathi et al., 2019). Cells were plated at an initial density of 3 × 10^4^ cells/mL and subsequently exposed to a range of TG concentrations (25, 50, 100, 200, and 400 μg/mL) (Yuanye Bio-Technology, Shanghai, China). A negative control group without any drug treatment was included, while a positive control group was treated with 200 μM HU (Sigma, United States). HU has been demonstrated to increase HbF levels (Steinberg, 2020) in both K562 cells and adult ErPCs (Zhu et al., 2014), and it is utilized in the treatment of β-thalassemia patients (Sales et al., 2021; Gambari et al., 2024). It is noted that 200 μM HU is considered the optimal concentration for inducing HbF production. The cells were incubated at 37°C in a humidified environment with 5% CO_2_ for 1–5 days.

### 2.3 CD34^+^ cell isolation and culture

CD34^+^ cells were isolated from umbilical cord blood samples collected from three voluntary donors at the Department of Medical Genetics, First People’s Hospital of Yunnan Province. All participants provided informed consent, and the study protocol was approved by the hospital’s Ethics Committee. Fresh cord blood was obtained using sodium heparin collection bags (Nigale, China) to ensure proper anticoagulation. Following the manufacturer’s guidelines, the blood was diluted with phosphate-buffered saline (PBS) at a 1:1 ratio. The mononuclear cell fraction was isolated using Lymphoprep™ (STEMCELL Technologies, Canada), and subsequent purification of CD34^+^ cells was performed using the CD34 MultiSort Kit for humans (Miltenyi Biotec, Germany) for monocyte sorting (Eghbali et al., 2023).

The cultivation of CD34^+^ cells was performed according to established protocols (Zuccato et al., 2021). In the initial phase (days 1–6), cells were cultured in Iscove’s Modified Dulbecco’s Medium (IMDM) supplemented with 100 ng/mL Stem Cell Factor (SCF; Proteintech, United States), 100 ng/mL Fms-like Tyrosine Kinase-3 (Flt-3; Proteintech, United States), 20 ng/mL Interleukin-3 (IL-3; Proteintech, United States), as well as 100 U/mL penicillin and 100 mg/mL streptomycin (Gibco, United States). In the subsequent phase (days 7–14), the cells were maintained in IMDM containing 2 U/mL Erythropoietin (Proteintech, United States), 50 ng/mL SCF (Proteintech, United States), 100 U/mL penicillin, and 100 mg/mL streptomycin (Gibco, United States). On the 7th day of culture, varying concentrations of TG (25 μg/mL, 50 μg/mL, 100 μg/mL, 200 μg/mL, and 400 μg/mL) were added to the culture medium, with 200 μM HU was used as a positive control (Ali et al., 2021), and no drug added as a negative control. At predetermined time points, samples were collected for phenotypic characterization, viability evaluation, and gene expression analysis.

### 2.4 *In vivo* study using townes model mice and ethical approval

Townes model mice, purchased from The Jackson Laboratory, were used to further validate the *in vivo* capability of TG to induce γ-globin gene expression and HbF production. The genetic background of these Townes model mice is B6; 129-Hbb^tm2(HBG1,HBB*)Tow^/Hbb^tm3(HBG1,HBB)Tow^Hba^tm1(HBA)Tow^/J (The Jackson Laboratory, United States, RRID:IMSR_JAX:013071), which harbor multiple human hemoglobin knock-in genes replacing endogenous mouse genes. All animal studies were approved by the Animal Use and Care Ethics Committee of the Laboratory Animal Center at Kunming University of Science and Technology. The mice were housed under a semi-natural light cycle (12:12 h light) in an environment maintained at 20°C–25°C and 55%–65% relative humidity (Steel et al., 2024). They were kept in cages with sawdust bedding and had *ad libitum* access to standard mouse chow and water.

### 2.5 *In vivo* analysis of treatment protocols

To evaluate the *in vivo* induction potential of TG, 6–8 week-old adult Townes model mice (18–24 g) were divided into seven groups (*n* = 3 per group). The groups received different doses of TG (50, 100, 200, 400, and 800 mg/kg/day). The in vivo TG dose range (50-800 mg/kg/day) was established based on previously published acute toxicity and safety data on Panax ginseng extracts and ginsenoside constituents (Carabin et al., 2000). HU at 200 mg/kg/day served as the po`sitive control, whereas 0.1% DMSO in saline was used as the negative control. Treatments were administered intraperitoneally for 2 weeks. Fifteen days post-treatment, all mice were euthanized by cervical dislocation under anesthesia (ketamine at 70 mg/kg and xylazine at 10 mg/kg) (Qiu et al., 2014). Blood samples were obtained via cardiac puncture into EDTA-coated tubes for flow cytometric analysis, and bone marrow was harvested from the hind limbs for total RNA extraction.

### 2.6 Cell viability and toxicity assessment

Cell viability and cytotoxicity were assessed utilizing CCK-8 assays, commonly employed to evaluate cell proliferation and cytotoxic effects by measuring color changes in the WST-8 reagent (Xia et al., 2017). In this study, the test culture was mixed with a 10% CCK-8 solution (Meilunbio, China) and incubated at 37°C for 45 min. The absorbance was measured at 450 nm utilizing an ELISA plate reader (Bio-Rad, iMark, United States). Cell viability was determined by calculating the percentage using the following formula: [(experimental well absorbance - blank well absorbance)/(control well absorbance - blank well absorbance)] × 100%. The inhibition rate was determined by: [(control well absorbance - experimental well absorbance)/(control well absorbance - blank well absorbance)] × 100%.

### 2.7 Hemoglobin (Hb) assessment

#### 2.7.1 Benzidine-peroxide hemoglobin staining

The erythroid differentiation of K562 cells can be assessed by utilizing the hemoglobin-binding affinity of a benzidine-peroxide solution (Lampronti et al., 2009). In brief, cells were combined with a freshly prepared benzidine-peroxide solution containing 0.2% benzidine (Merck, Germany) in 0.5 M acetic acid (Sigma, United States) along with 10 µL of 30% H_2_O_2_ (Sigma, United States) (Salvatori et al., 2009). Subsequently, the cells were incubated in the dark for 5 min. Following the incubation, the cells were examined under an optical microscope (Nikon, Japan), and the benzidine-positive cells were quantified.

#### 2.7.2 Total Hb determination

To quantitatively analyze cellular Hb levels, the Hemoglobin Assay Kit was employed (Sigma, United States). On the fourth day of treatment, an equal number of cells were collected and then washed twice with cold PBS. Subsequently, Hb was converted to a colorimetric product, which was measured at 400 nm. Optical density (OD) values were obtained using an ELISA plate reader (Bio-Rad, iMark, United States) with 400 nm excitation. Hb levels were calculated as [(sample well OD - blank well OD)/(standard well OD - blank well OD)] × 100 mg/dL × dilution factor.

### 2.8 HbF analysis by flow cytometry

On day 4 of treatment, the cells were harvested, washed twice with ice-cold PBS, fixed with 4% paraformaldehyde (PFA; Servicebio Wuhan, China), and permeabilized with 0.1% Triton X-100 (Sigma, United States) for 4 min. Subsequently, the cells were resuspended in 100 µL PBS and incubated in the dark for 20 min with 5 µL of MHFH04 PE-conjugated monoclonal anti-HbF antibody (Life Technologies, United States). Following three washes with PBS and centrifugation at 300 × g for 5 min, the stained cells were resuspended in 0.5 mL PBS and analyzed using a BD FACSCII flow cytometer (Becton Dickinson, United States) with FlowJo version 10.7.2.

### 2.9 Immunocytochemistry staining

On day 4 of treatment, cells were collected, washed twice in ice-cold PBS, fixed with 4% PFA, and permeabilized using 0.1% Triton X-100 for 4 min. The cells were then resuspended in 100 µL PBS and incubated in the dark for 20 min with 5 µL of MHFH04 PE-conjugated monoclonal anti-HbF antibody (Life Technologies, United States) (Fathi et al., 2022). After three PBS washes and centrifugation at 300 × g for 5 min, the cells were resuspended in 300 µL PBS and imaged using a confocal laser scanning microscope (Zeiss, Germany). The resulting images were processed with ZEN software (Zeiss, Germany), and the average fluorescence intensity per cell was quantified using ImageJ software.

### 2.10 RNA isolation and quantitative real-time PCR (qRT-PCR) analysis

Total RNA was isolated from cells on day 4 using the RNAprep FastPure Animal Tissue/Cell Total RNA Isolation Kit (Tsingke, China). The RNA concentration was measured with a Nanodrop 8000 (Thermo Scientific, United States), and its integrity was confirmed via agarose gel electrophoresis. Next, 1 μg of total RNA was reverse transcribed into first-strand cDNA using the HiScript III RT SuperMix for qPCR (+gDNA wiper) kit (Tsingke, China). The β-actin primers were 5′-CCT​GAA​CCC​CAA​GGC​CAA​CC-3′ and 5′-CAG​GGA​TAG​CAC​AGC​CTG​GA-3'. The human γ-globin gene primers were 5′-GGG​GCA​AGG​TGA​ATG​TGG​AAG​A-3′ and 5′-CAT​GAT​GGC​AGA​GGC​AGA​GGA​C-3'.

To prepare a 20 μL reaction system, the 2×TSINGKE® Master qPCR Mix (SYBR) (Tsingke, China) was utilized. The system included 10 μL of 2× ChamQ Universal SYBR qPCR Master Mix, 0.4 nM of each gene-specific forward and reverse primer, and 100 ng of cDNA template. PCR amplification was carried out using an ABI 7300 qPCR instrument (Thermo Fisher, United States). The qPCR protocol began with an initial denaturation at 95°C for 60 s, followed by 40 cycles consisting of 95°C for 10 s (denaturation) and 60°C for 30 s (annealing) with fluorescence signal collection at 60°C. The specificity of the PCR products was verified by analyzing the melting curves.

### 2.11 H&E staining of mouse liver tissue

Mouse liver tissues were fixed in 4% paraformaldehyde (Servicebio, China) and subsequently dehydrated through a graded ethanol series. The tissues were then cleared in xylene (Shanghai, China) and embedded in paraffin (Sigma-Aldrich, United States). Once the paraffin blocks had solidified, they were trimmed and sectioned into 5 μm thick slices. The sections were deparaffinized in xylene, rehydrated through a graded ethanol series, and stained with hematoxylin and eosin (Servicebio, China). Finally, the slides were mounted with neutral resin (Servicebio, China). Tissue morphology was examined under a microscope, and images were captured using a slide scanner.

### 2.12 Statistical analysis

Statistical analyses were performed using GraphPad Prism 6 (GraphPad Software, United States). Data are presented as mean ± standard deviation (SD). Group differences were evaluated by one-way ANOVA followed by the Student–Newman–Keuls test. Statistical significance was denoted as follows: *P < 0.05, **P < 0.01, and ***P < 0.001.

## 3 Results

### 3.1 Analysis of the major components of TG

**FIGURE 1:**
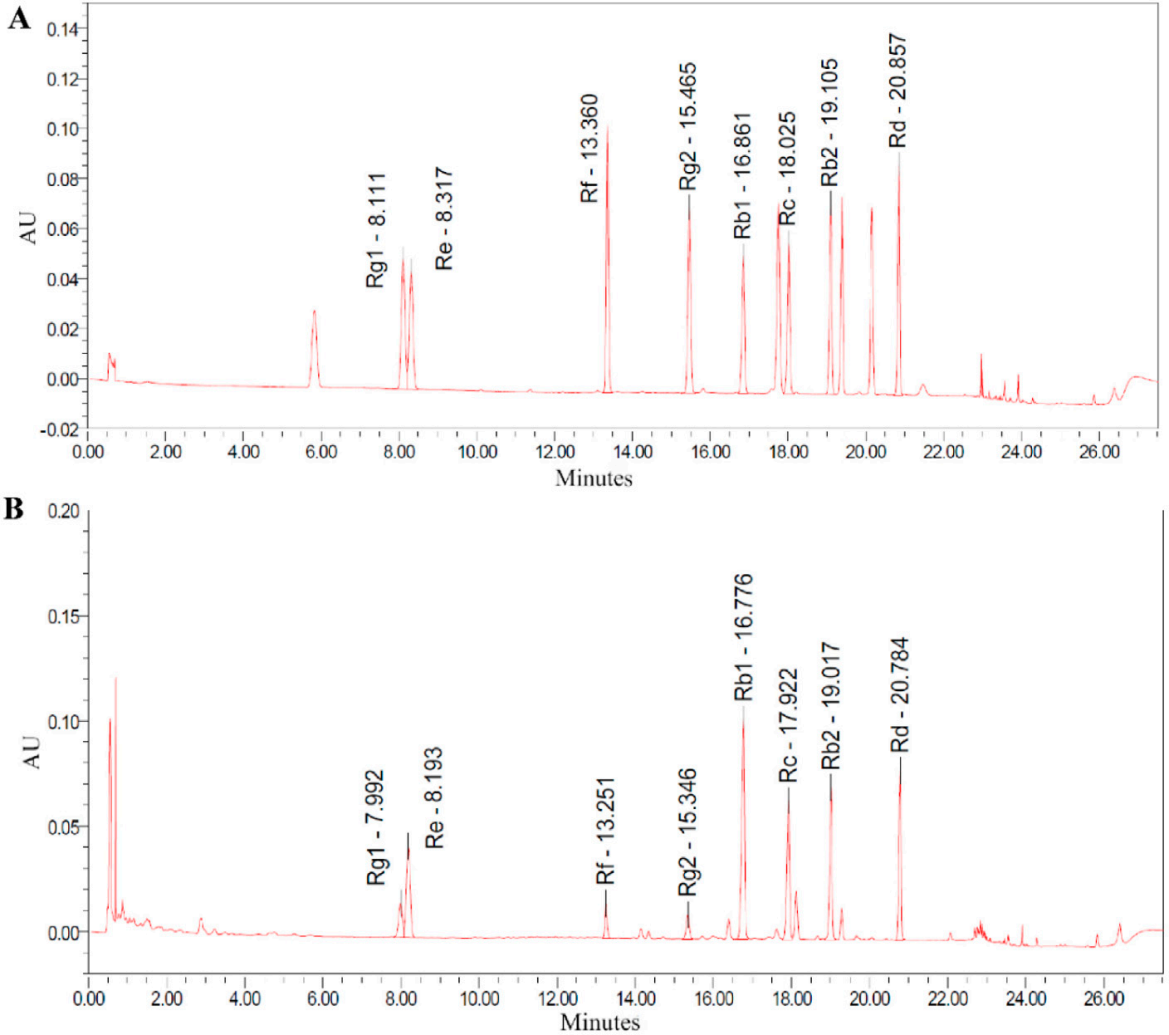
HPLC chromatograms recorded at 203 nm. **(A)** Standard ginsenosides. **(B)** TG.

HPLC analysis of TG confirmed the presence of key ginsenosides. Chromatographic peaks corresponded to known standards, specifically ginsenosides Rg1, Re, Rf, Rg2, Rb1, Rc, Rb2, and Rd, as shown in Figure 1A. The relative abundances of these ginsenosides in TG were quantified, with concentrations presented in Figure 1B and tabulated in Table 1. The respective ginsenoside contents were as follows: Rg1 constituted 3.567%, Re 10.307%, Rf 1.840%, Rg2 1.690%, Rb1 23.925%, Rc 12.377%, Rb2 11.391%, and Rd 10.450%. Additionally, smaller amounts of other ginsenosides and phytochemical components were identified in TG.

**TABLE 1:** Analysis of ginsenoside content in TG.

Ginsenoside components	Retention time (minutes)	Area (microvolt × second)	Height (microvolt)	Contents (%)
Rg1	7.992	110,735	16,344	3.567
Rc	8.193	299,340	43,375	10.307
Rf	13.251	68,566	16,718	1.840
Rg2	15.346	63,427	11,582	1.690
Rb1	16.776	562,305	104,396	23.925
Rc	17.922	321,586	65,876	12.377
Rb2	19.017	301,337	72,186	11.391
Rd	20.784	291,791	80,405	10.450

### 3.2 Accumulation of total hemoglobin mediated by TG in K562 cells

**FIGURE 2:**
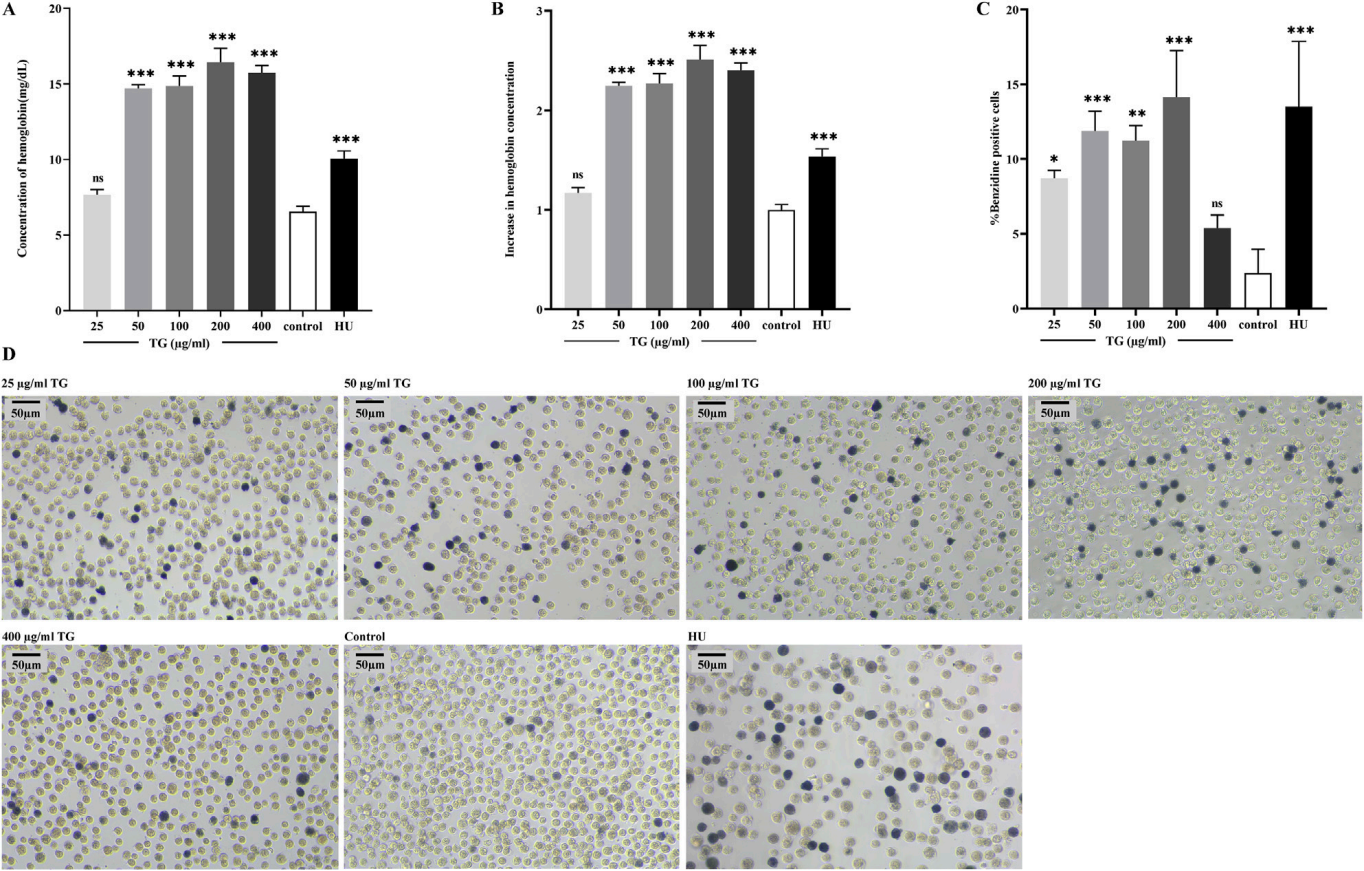
TG-induced total hemoglobin synthesis in K562 cells. **(A)** Total hemoglobin content was quantified in K562 cells following TG treatment. **(B)** A fold increase in total hemoglobin levels was observed in TG-treated cells compared to the untreated control. **(C)** Proportion of benzidine-positive cells. **(D)** Representative photomicrographs (×20 objective) illustrate hemoglobinization in K562 cells. Cells were treated with various concentrations of TG, 200 μM HU, or left untreated; benzidine staining was used to highlight hemoglobin-positive cells.

Following a 4-day TG treatment on K562 cells, hemoglobin (Hb) levels were quantitatively assessed. As shown in Figure 2A, TG significantly enhanced Hb synthesis: cells exposed to 50, 100, and 200 μg/mL TG exhibited substantial increases in Hb levels to 14.71 ± 0.22, 14.86 ± 0.66, and 16.44 ± 0.92 mg/dL, respectively, all p < 0.0001, compared to the control’s 6.55 ± 0.35 mg/dL. The Hb concentration in cells treated with the optimal erythroid induction dose of 200 µM HU was 10.07 ± 0.50 mg/dL (p < 0.0001). Hb fold changes under various treatments are depicted in Figure 2B, demonstrating a dose-dependent rise in TG-induced Hb levels.

Benzidine-H_2_O_2_ staining—a technique utilizing the peroxidase activity intrinsic to the heme component of Hb—further substantiated red cell differentiation by identifying hemoglobinized cells (Mischiati et al., 2004). This staining showed a substantial increase in benzidine-positive cells upon treatment with 50, 100, and 200 μg/mL TG, detailed in Figures 2C,D. Specifically, the proportions of benzidine-positive cells surged to 11.88% ± 1.31%, 11.25% ± 1.00%, and 14.14% ± 3.12%, respectively, each significantly higher than the control’s 2.38% ± 1.59%. In the group treated with 200 μM HU, benzidine-positive cells comprised 13.51% ± 4.36% of the total population.

Additionally, as shown in Figure 2D, K562 cells exposed to 200 µM HU exhibited a significant increase in cell size (Iftikhar et al., 2019), a phenomenon not observed in TG-treated cells, where no significant morphological or size alterations were noted. Collectively, these findings underscore that TG can selectively boost Hb levels in K562 cells without adversely affecting cell morphology. Results are presented as mean ± SD for a sample size of three.

### 3.3 TG increased the production of HbF in K562 cells

**FIGURE 3:**
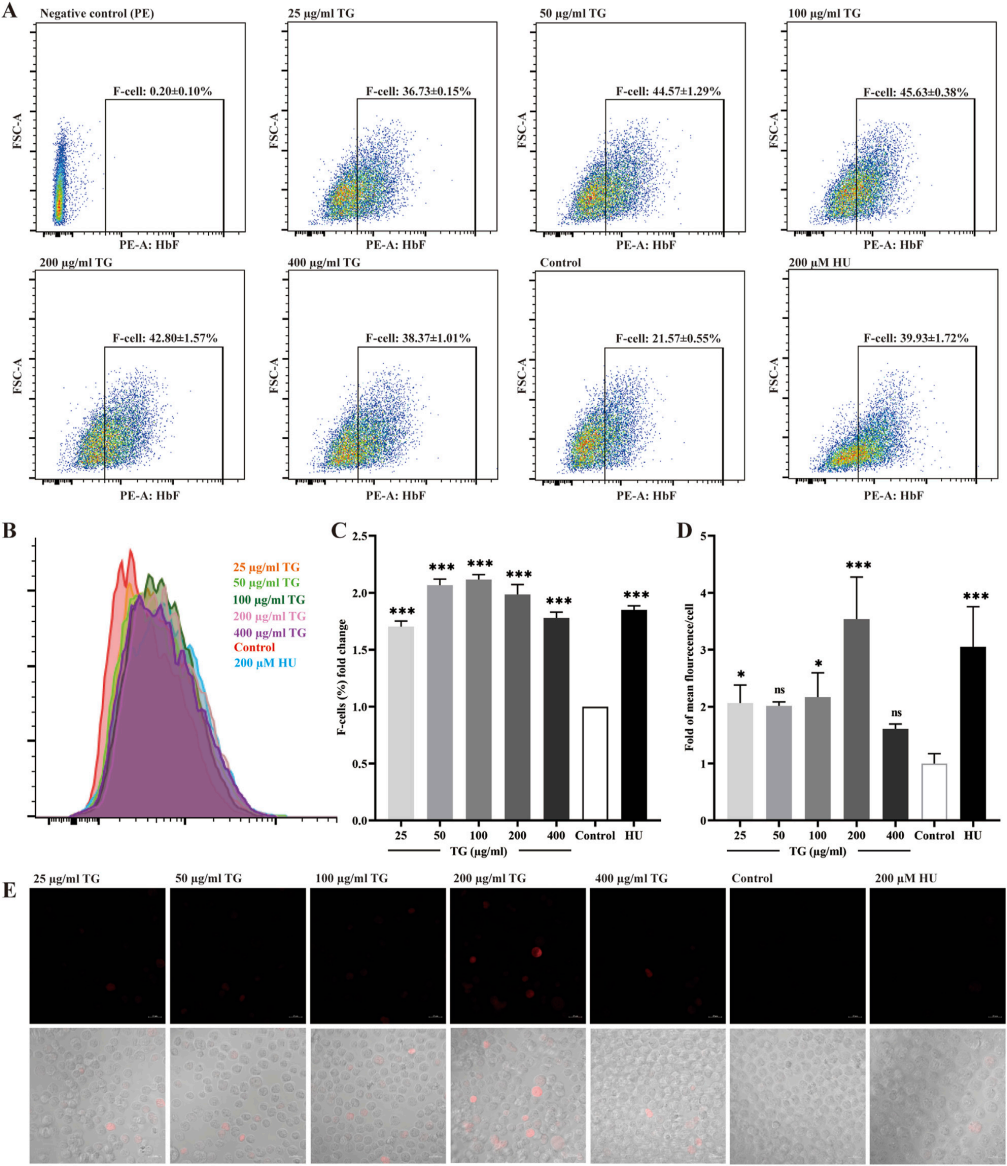
Assessment of HbF expression in K562 cells treated with TG. **(A)** Flow cytometry analysis shows the F-cell population after TG treatment. **(B)** An overlay histogram illustrates the distribution of cells positively stained with the anti-HbF antibody. **(C)** The proportional changes in F cells relative to the negative control group. **(D)** Histograms display the MFI of HbF-PE for each treatment group. **(E)** Immunofluorescent images (×40 magnification) demonstrate HbF labeling in TG-treated cells.

Figures 3A–C illustrate a significant increase in the proportion of F cells, which are defined by their expression of HbF, following treatment of K562 cell cultures with various concentrations of TG. At doses of 50, 100, and 200 μg/mL TG, the proportions of F-cells were 44.57% ± 1.29%, 45.63% ± 0.38%, and 42.80% ± 1.57%, respectively. These values represent fold increases of 2.07 ± 0.05, 2.12 ± 0.43, and 1.99 ± 0.09 compared to the untreated control group’s baseline F-cell proportion of 21.57% ± 0.55%. Under the influence of 200 μM HU, the F-cell percentage increased to 39.93% ± 1.72%, reflecting a 1.85 ± 0.04-fold enhancement.

Using human anti-HbF monoclonal antibodies for immunocytochemical staining, Figures 3C,D demonstrate a marked upregulation of HbF expression in K562 cells treated with 100 and 200 μg/mL TG, as indicated by the presence of red fluorescence. The fluorescence intensity per cell, measured in arbitrary units (AU), was quantitatively analyzed. TG treatment at 100 and 200 μg/mL significantly increased the mean fluorescence intensity (MFI) intensity of HbF to 20.37 ± 2.92 AU and 19.70 ± 4.17 AU, respectively, compared to 4.07 ± 2.49 AU in the untreated control group. Notably, the positive control group treated with 200 µM HU exhibited a higher average HbF fluorescence intensity of 28.37 ± 3.33 AU.

Furthermore, Figure 3D confirms that while 200 µM HU caused a significant increase in K562 cell size, TG treatment did not materially alter cell dimensions or morphology. Overall, a TG concentration range of 50–100 μg/mL was effective in elevating HbF levels in K562 cells. These observations are rigorously documented, with data expressed as mean ± standard deviation, based on triplicate assays.

### 3.4 TG upregulated γ-globin gene expression in K562 cells

**FIGURE 4:**
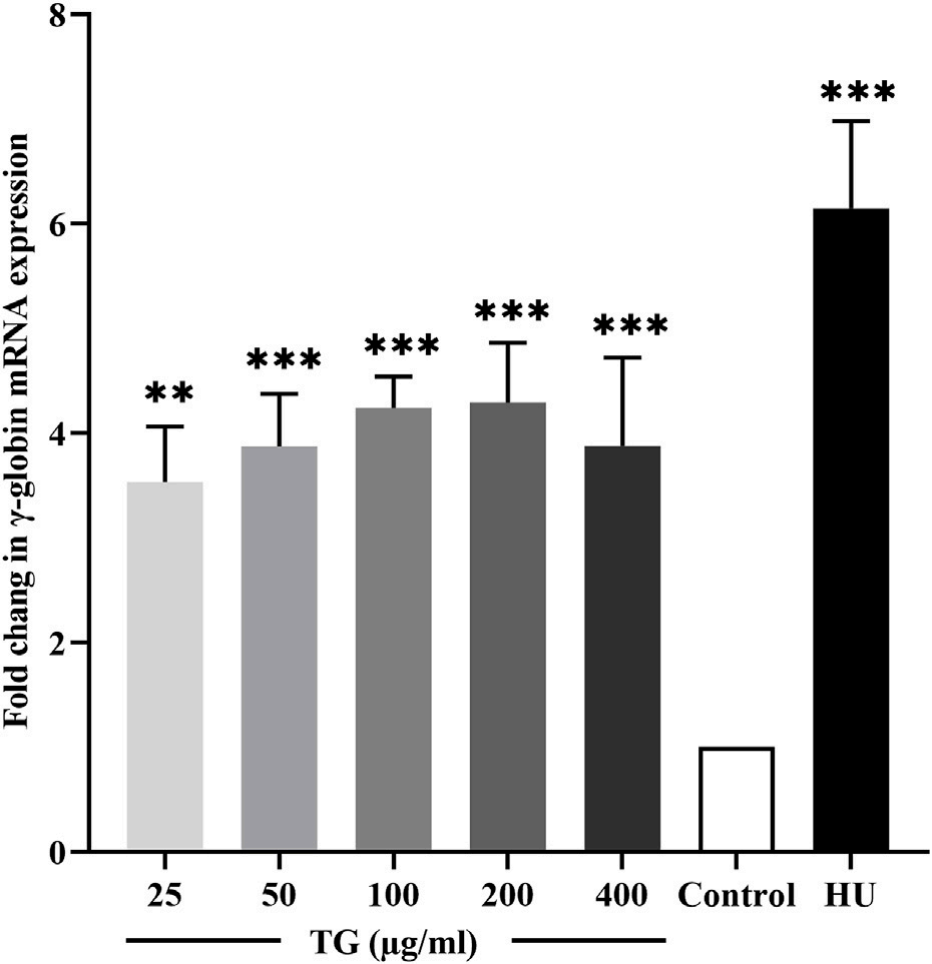
Quantitative RT-PCR analysis showing the fold change in γ-globin mRNA expression in TG-treated K562 cells compared to the untreated control. Data represent mean ± SD from triplicate experiments (*n* = 3).

The transcriptional activity of the γ-globin gene in response to TG was evaluated using RT-qPCR. As shown in Figure 4, K562 cells treated with TG at concentrations of 50–200 μg/mL exhibited a significant induction of HBG transcription. Specifically, HBG expression increased by 3.87 ± 0.50-fold at 50 μg/mL, 4.23 ± 0.30-fold at 100 μg/mL, and 4.29 ± 0.57-fold at 200 μg/mL, with all increases being statistically significant (p < 0.001 and p < 0.01) compared to untreated controls. In contrast, cells treated with 100 µM HU showed a more pronounced 6.14 ± 0.84-fold increase in HBG mRNA levels (p < 0.001).

### 3.5 The effect of TG on the growth of K562 cells

**FIGURE 5:**
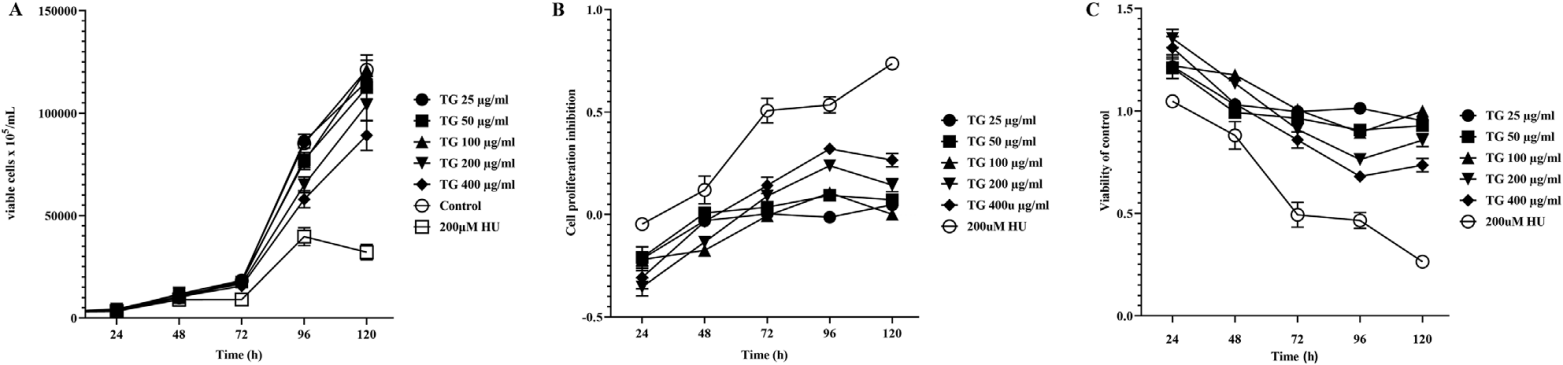
Influence of TG on K562 cell proliferation and viability. **(A)** Growth curve showing the proliferation of K562 cells over time under TG treatment. **(B)** Inhibition rates of K562 cell proliferation in response to TG and HU treatments compared to the negative control. **(C)** Cell viability of K562 cells treated with TG, assessed relative to the negative control group. Data represent mean ± SD from triplicate experiments (*n* = 3).

Figure 5 illustrates the impact of varying TG concentrations (25–400 μg/mL) on the proliferation dynamics of K562 cells. Panels 5A-C present the results of cell viability and growth inhibition rates as determined by the CCK-8 assay, conducted following TG administration across a range of doses for incubation periods extending from 1 to 5 days. The data reveal a clear dose- and time-dependent cytostatic effect of TG on K562 cells, with higher concentrations and prolonged exposure leading to greater inhibition of cell proliferation. Notably, compared to the response after treatment with the standard erythroid differentiation inducer, 200 μM HU, TG shows a slight reduction in K562 cell proliferation rates at various dosages. However, overall cell viability was not significantly compromised, suggesting a cytostatic rather than cytotoxic effect of TG on these cells.

### 3.6 Accumulation of total hemoglobin mediated by TG in ErPCs

In this study, written informed consent was obtained from three individuals at the Department of Medical Genetics, First People’s Hospital of Yunnan Province. CD34^+^ progenitor cells were isolated from the umbilical cord blood of healthy newborns and cultured along the erythroid lineage to differentiate into ErPCs. During differentiation, the cells were treated with TG at concentrations ranging from 25 to 400 μg/mL. After the culture period, the cells were harvested for quantitative assessment of Hb levels.

**FIGURE 6:**
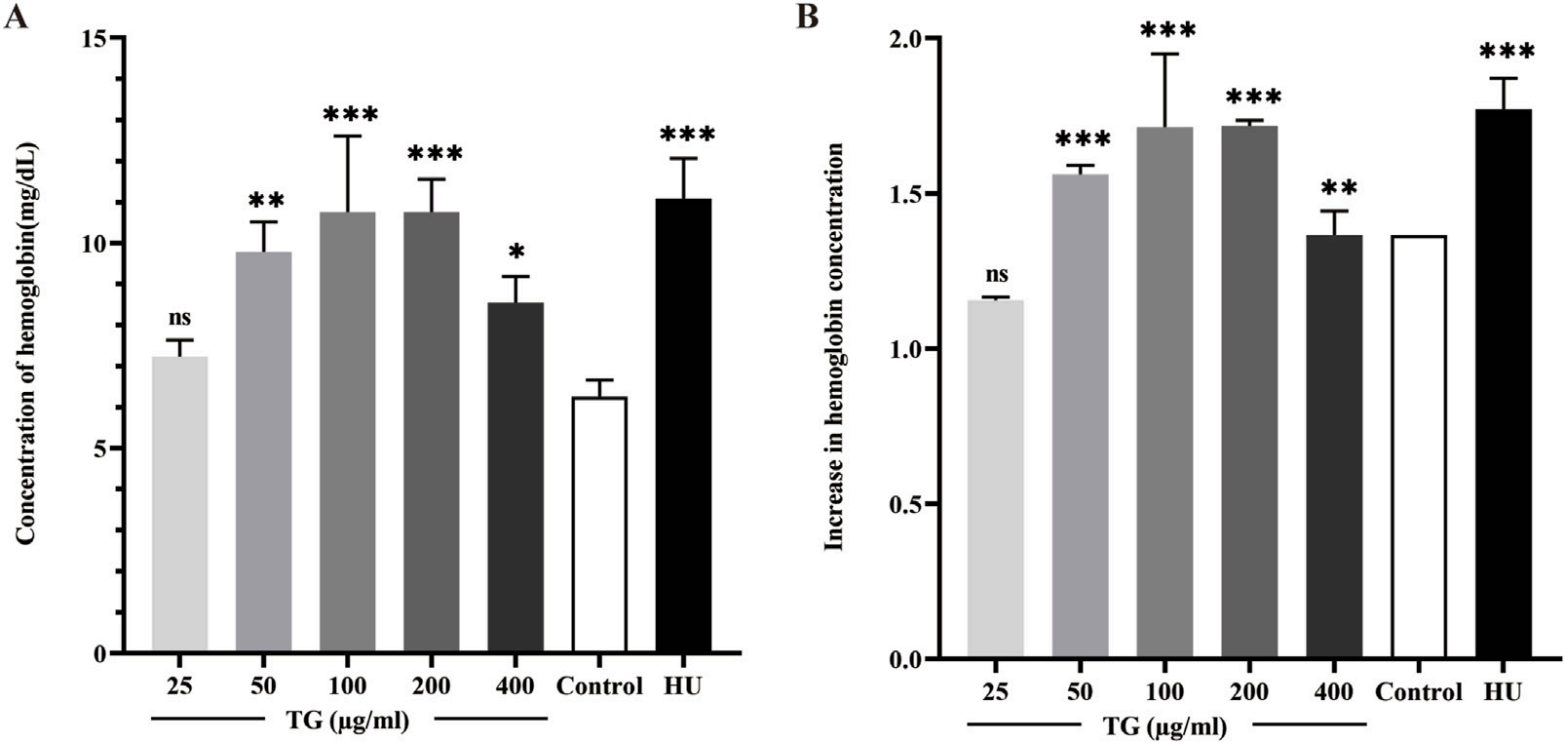
Analysis of Hb expression in ErPCs treated with TG. **(A)** Hemoglobin concentration in ErPCs after TG treatment, expressed in mg/dL. **(B)** Fold change in hemoglobin levels relative to the negative control. Data represent mean ± SD from three independent experiments (*n* = 3).

The quantitative analysis of Hb levels, illustrated in Figures 6A,B, indicates that treatment with TG increases Hb content in ErPCs. The data show that ErPCs subjected to 50, 100, and 200 μg/mL of TG exhibited significant elevations in Hb concentrations, measuring 9.78 ± 0.73, 10.75 ± 1.85, and 10.75 ± 1.85 mg/dL, respectively. These values contrast sharply with the control group’s 6.25 ± 0.41 mg/dL, with all TG treatment groups reaching statistical significance (p < 0.0001). Additionally, Hb levels in cells treated with 200 µM HU were higher at 11.09 ± 0.98 mg/dL (p < 0.0001). Figure 6B presents the fold increase in Hb across different treatment conditions, clearly demonstrating a TG dose-dependent enhancement in Hb levels.

### 3.7 TG upregulates HbF expression in ErPCs

**FIGURE 7:**
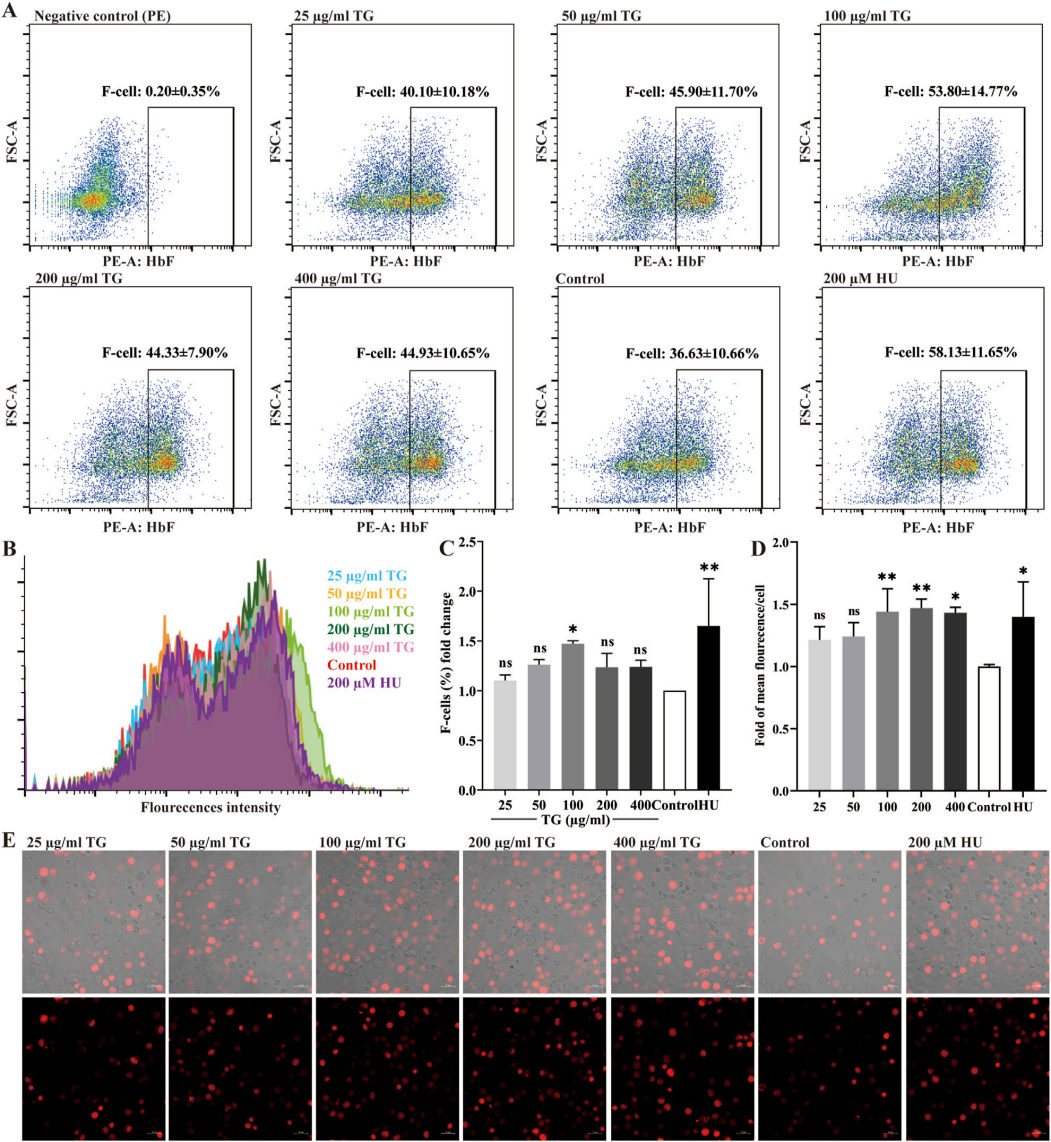
Assessment of HbF expression in ErPCs treated with TG. **(A)** Flow cytometry analysis illustrating the F-cell population following TG treatment. **(B)** Overlay histogram displaying the distribution of cells positively stained with the anti-HbF antibody. **(C)** Proportional changes in the F-cell population relative to the negative control group. **(D)** Histograms presenting the MFI of HbF-PE across different treatment groups. **(E)** Immunofluorescent images (×40 magnification) highlighting HbF labeling in TG-treated cells.

ErPCs were exposed to TG at concentrations ranging from 25 to 400 μg/mL to evaluate its impact on intracellular HbF expression. After culture, flow cytometry and immunofluorescence analyses were performed to quantify HbF levels, as shown in Figures 7A–C. Treatment with TG significantly increased the proportion of HbF-positive ErPCs compared to the negative control. Specifically, ErPCs treated with 50–200 μg/mL TG showed the most significant increases in HbF percentage, reaching 45.90% ± 11.70%, 53.80% ± 14.77%, and 44.33% ± 7.90%, corresponding to 1.26 ± 0.05-fold, 1.47 ± 0.03-fold, and 1.24 ± 0.14-fold increases, respectively. In parallel, ErPCs treated with 200 μM HU exhibited a 1.65 ± 0.47-fold enhancement in HbF expression, with a percentage of 58.13% ± 11.65%.

Immunofluorescence analysis (Figures 7D,E) further supports these findings, revealing enhanced HbF expression in ErPCs treated with TG. The MFI for the 100 and 200 μg/mL TG groups were notably increased, registering at 17.64 ± 2.24 and 17.98 ± 0.89, respectively, compared to an MFI of 12.24 ± 0.19 in the negative control. In contrast, the 200 μM HU treatment group displayed an MFI of 17.12 ± 3.44. In summary, the experimental data strongly indicate that TG treatments across the tested concentrations significantly increases both the proportion of HbF-positive cells and the overall fluorescence intensity in ErPCs, with the 100–200 μg/mL dosage range exhibiting the greatest efficacy.

### 3.8 TG upregulated γ-globin gene expression in normal human ErPCs

**FIGURE 8:**
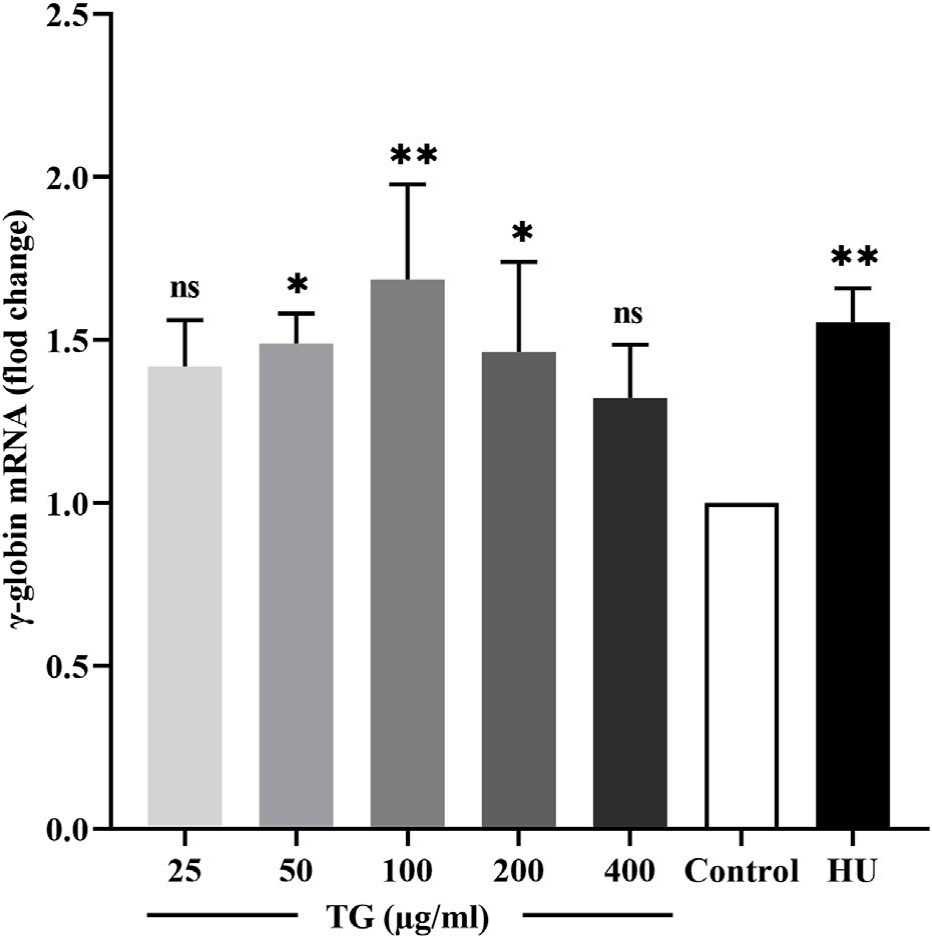
Impact of TG on γ-globin gene expression in ErPCs. Data are presented as mean ± SD (*n* = 3). Relative expression levels of γ-globin mRNA were normalized against β-actin RNA, are shown.

The effect of TG on γ-globin gene transcription in ErPCs was quantitatively evaluated using RT-qPCR. As shown in Figure 8, treatment with 100 and 200 μg/mL TG resulted in significant increases in γ-globin mRNA expression, with fold changes of 1.69 ± 0.29 (p < 0.05) and 1.46 ± 0.27 (p < 0.01), respectively, relative to the negative control. Moreover, cells treated with 200 μM HU exhibited a 1.55 ± 0.10 (p < 0.01)-fold increase in γ-globin expression.

These results underscore the significant role of TG in elevating HbF levels in ErPCs. The observed enhancement in HbF aligns with the upregulation of γ-globin mRNA, suggesting a potential mechanism wherein TG may potentiate HbF synthesis. This mechanism likely involves the coordinated upregulation of fetal globin gene transcription, proposing a promising therapeutic avenue for conditions ameliorated by increased HbF production.

### 3.9 TG induces *in vivo* HbF expression

To further investigate the regulatory effect of TG on HbF expression *in vivo*, Townes model mice were used as the animal model. The mice received intraperitoneal injections of TG for 14 consecutive days. At the end of the treatment period, blood samples were collected via cardiac puncture and analyzed by flow cytometry to determine the proportion of HbF-positive cells.

**FIGURE 9:**
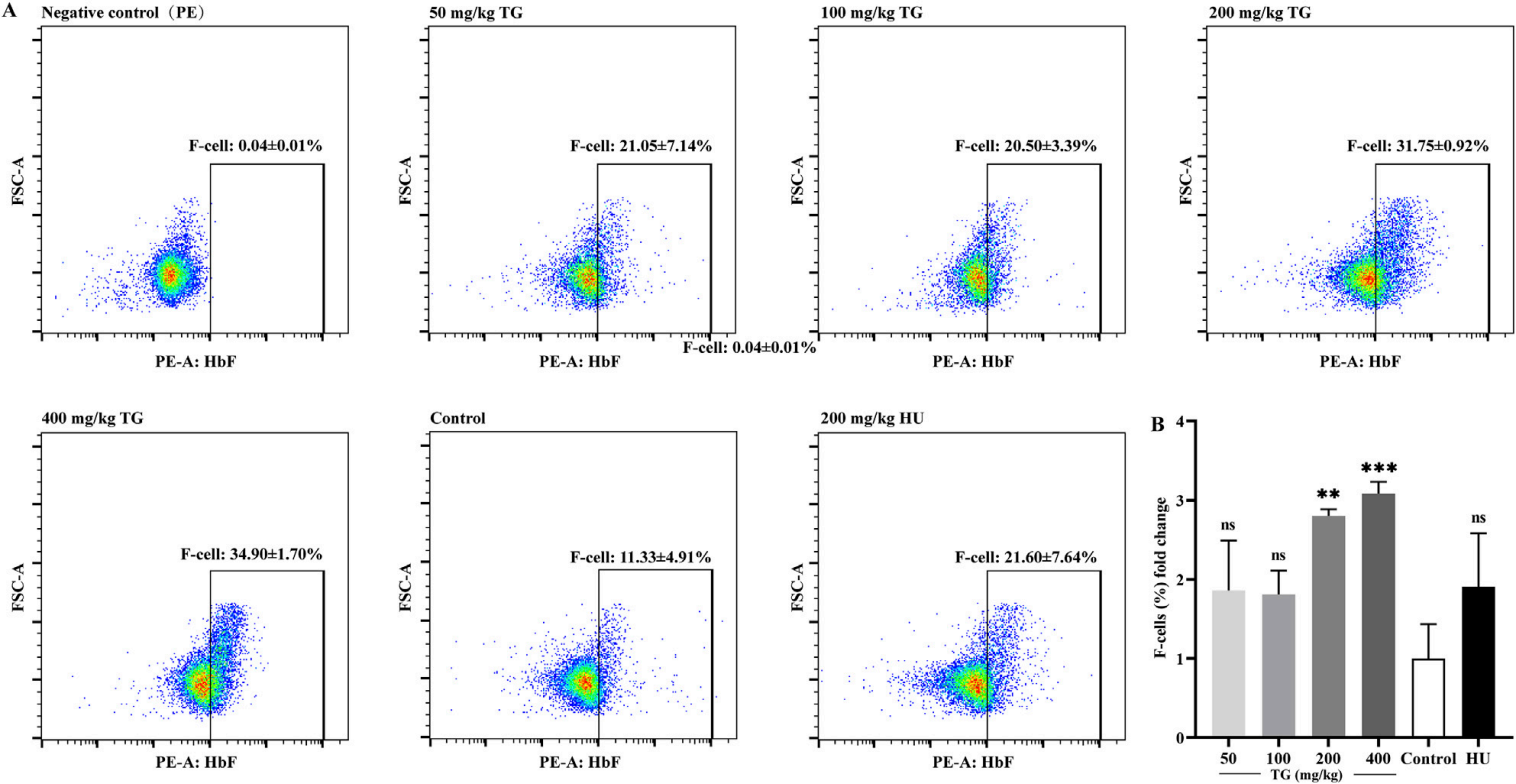
FACS analysis of the impact of TG on HbF expression in mice. **(A)** Proportion of F-cells in each treatment group. **(B)** Fold change in the proportion of F cells relative to the negative control group.

As shown in Figure 9, TG effectively promoted HbF production *in vivo*. Specifically, at doses of 200 and 400 mg/kg, the proportions of F cells reached 31.75% ± 0.92% and 34.90% ± 1.70%, respectively, compared to 11.33% ± 4.91% in the negative control group. These represent 2.80 ± 0.08- and 3.08 ± 0.15-fold increases over the control. In contrast, the positive control group treated with 200 mg/kg HU exhibited an F-cell proportion of 21.60% ± 7.64%, corresponding to a 1.91 ± 0.67-fold increase relative to the negative control group.

### 3.10 TG upregulated γ-globin gene expression *in vivo*

**FIGURE 10:**
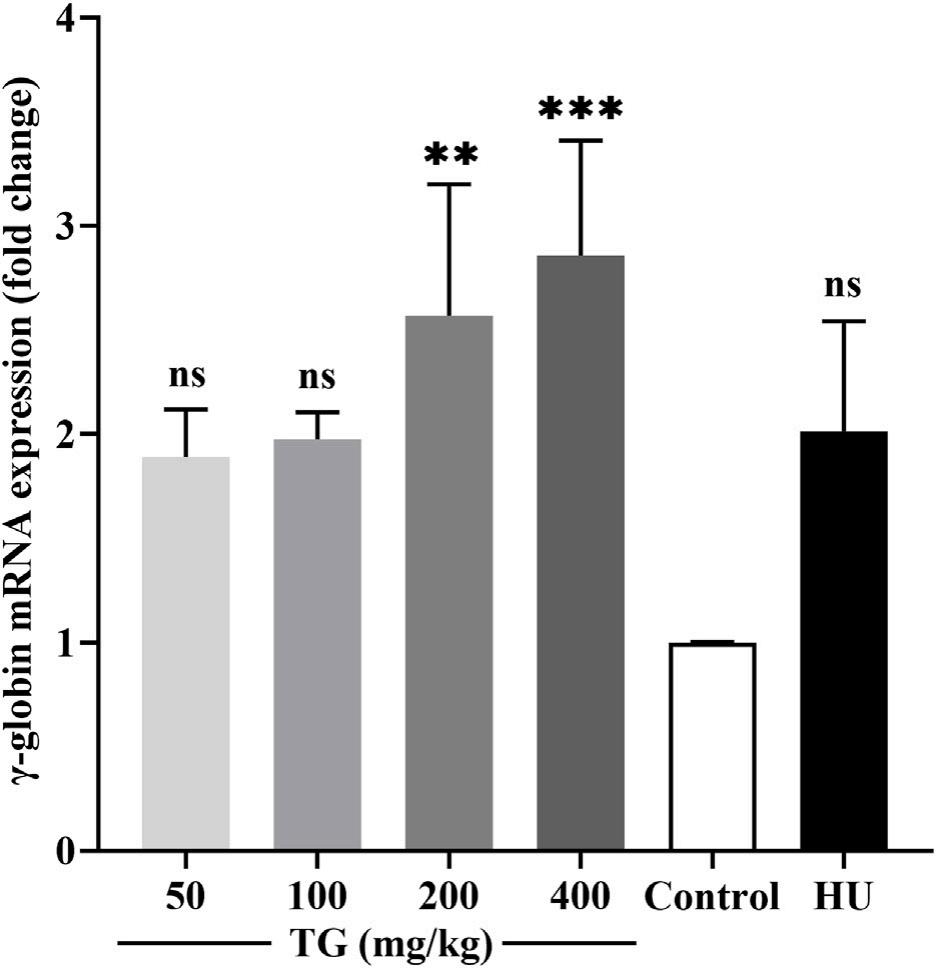
Impact of TG on γ-globin gene expression *in vivo*. Relative expression levels of γ-globin mRNA in mouse bone marrow, normalized to β-actin mRNA, as determined by RT-qPCR. Data are presented as mean ± SD (*n* = 3).

As shown in Figure 10, RT-qPCR results demonstrate that TG significantly enhances γ-globin gene expression in mice. Specifically, treatment with TG at doses of 200 mg/kg and 400 mg/kg increased γ-globin mRNA levels by 2.57 ± 0.63-fold and 2.86 ± 0.55-fold, respectively, compared to the negative control. In contrast, the HU treatment group exhibited a 2.01 ± 0.53-fold increase in γ-globin mRNA expression relative to the negative control.

### 3.11 Histopathological analysis of mouse liver to assess TG toxicity

**FIGURE 11:**
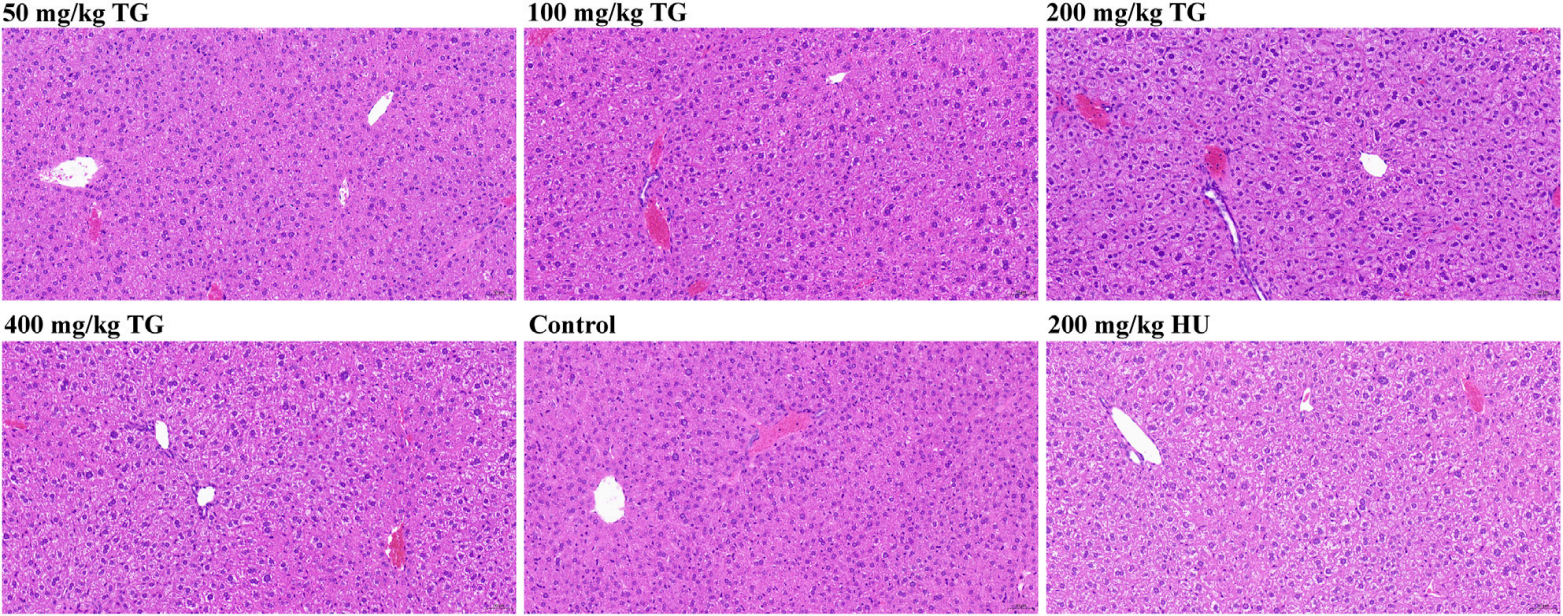
Histopathological analysis of mouse liver following TG treatment.

To investigate the potential hepatotoxicity of TG, we conducted histopathological examinations on liver sections from Townes model mice following 14 days of intraperitoneal injections with varying doses of TG, saline (control), and HU as a positive control. As shown in Figure 11, in the control group, many hepatocytes exhibited edema and cytoplasmic swelling, with loose, pale staining cytoplasm, compressed sinusoidal spaces, and unclear hepatic cord structures. No significant inflammatory cell infiltration was observed. Similar changes were noted in TG-treated groups, with hepatocyte edema, swelling, and mild congestion observed at doses of 50, 100, 200, and 400 mg/kg TG. The 200 mg/kg dose showed widespread ballooning of hepatocytes with sparse or finely reticulated cytoplasm. In the HU-treated group, hepatocyte edema and swelling were also observed, but no significant inflammatory infiltration was noted in any group.

Histopathological changes were mild and consistent across TG-treated, control, and HU-treated groups. Hepatocyte edema and swelling were present, but no severe damage or significant inflammation was detected, indicating that TG has a relatively low hepatotoxicity profile at the studied doses. This suggests that TG could be a safer alternative to HU for inducing HbF expression.

## 4 Discussion

This study demonstrates that TG significantly induce γ-globin expression and HbF production in both in vitro and in vivo models. In K562 cells and cord blood-derived erythroid progenitors, TG treatment elevated γ-globin mRNA levels and HbF-positive cells without adversely affecting erythroid differentiation. Importantly, in vivo administration of TG in Townes mice—a model expressing human globin genes including γ-globin—resulted in a marked increase in circulating F-cells. After 14 days of treatment, TG at 200–400 mg/kg nearly tripled F-cell percentages compared to controls, supporting its therapeutic potential as an HbF inducer.

Although the exact molecular mechanism of TG-induced γ-globin expression remains to be elucidated, existing studies provide some plausible insights. For instance, natural products with antioxidant properties (Feng et al., 2022; Mayuranathan et al., 2023)—including components of ginseng—have been associated with activation of NRF2, a transcription factor implicated in globin gene regulation through chromatin remodeling and oxidative stress responses (Zhu et al., 2017). Additionally, HbF induction by other agents has been linked to the downregulation of repressors such as BCL11A and ZBTB7A (Martyn et al., 2024). Whether TG affects these pathways directly remains unknown, but its bioactivity profile suggests possible modulation of erythroid-specific transcriptional networks. Further mechanistic studies will be essential to define the molecular underpinnings of its HbF-inducing effect.

Compared with HU, the only FDA-approved HbF inducer (Steinberg et al., 2014), TG showed comparable or even superior effects on γ-globin expression without observable cytotoxicity or adverse organ effects within the study duration. HU exerts its action partly through cytostatic effects and nitric oxide-mediated signaling, but also carries risks of bone marrow suppression, gastrointestinal symptoms, and potential long-term toxicity (Antonioli et al., 2012). In contrast, TG did not produce morphological abnormalities in bone marrow-derived cells, and liver histology revealed no significant damage. While these preliminary findings suggest a favorable safety profile, comprehensive toxicological assessment is required to confirm systemic tolerability.

Despite these encouraging results, several limitations must be acknowledged. First, our toxicity evaluation focused only on the liver. Other organs commonly affected in systemic toxicity—such as the kidney and spleen—were not assessed. Second, the mechanism underlying TG-induced HbF expression was not experimentally investigated; transcriptional repressors, epigenetic marks, or relevant signaling pathways were not measured. Third, while cord blood-derived progenitors were used, validation in erythroid cells from β-thalassemia patients remains essential to confirm clinical relevance. Lastly, although Townes mice carry human γ-globin genes, they do not recapitulate the ineffective erythropoiesis or anemia characteristic of β-thalassemia. Transgenic models with β-thalassemia mutations should be employed in future studies to more accurately evaluate therapeutic efficacy.

Future investigations should prioritize mechanistic studies to determine how TG modulates γ-globin expression. Particular attention should be given to its potential effects on epigenetic regulators or transcriptional repressors known to control the fetal-to-adult globin switch. Comprehensive toxicological assessments—including histological evaluation of the kidney, spleen, and bone marrow—are necessary to establish TG’s systemic safety profile. Additionally, validation using erythroid cells derived from β-thalassemia patients will help confirm translational relevance. Testing TG in genetically engineered mouse models of β-thalassemia will be critical to assess its effects on ineffective erythropoiesis and anemia. Together, these efforts will provide essential preclinical data to support future clinical development of TG as a novel therapy for β-hemoglobinopathies.

## 5 Conclusion

Our study demonstrates that TG is a promising inducer of HbF expression, with significant potential for treating β-thalassemia. Using both *in vitro* and *in vivo* models, we showed that TG effectively enhances Hb, HbF, and γ-globin gene expression. In K562 cells and ErPCs, TG treatment significantly upregulated these genes without affecting cell proliferation or morphology, positioning TG as a favorable alternative to HU. *In vivo* validation using Townes model mice further confirmed TG’s efficacy. Intraperitoneal administration of TG significantly increased the proportion of HbF-positive cells, supporting its potential clinical applicability.

In conclusion, TG is a potent inducer of HbF and represents a promising new therapeutic strategy for β-thalassemia. Our findings lay a strong foundation for future studies aimed at developing TG into a safer and more effective treatment option for patients with β-thalassemia.

## Data Availability

The original contributions presented in the study are included in the article/supplementary material, further inquiries can be directed to the corresponding author.
